# Supplementary material for: Developing a machine learning-based tool to extend the usability of the NICHD BPD Outcome Estimator to the Asian population
Source: PLoS One. 2022 Sep 16;17(9):e0272709. doi: 10.1371/journal.pone.0272709 (PMC9480997; doi:10.1371/journal.pone.0272709)
Supplement: S1 Table — (DOCX) [file pone.0272709.s002.docx]

**S1 Table. Model performance comparisons.**

| **Model** | **Support vector machine** | | **Random forest** | | **Ensemble** | |
| --- | --- | --- | --- | --- | --- | --- |
| **Data set** | **Train** | **Test** | **Train** | **Test** | **Train** | **Test** |
| *Postmenstrual Age 36 weeks* | | | | | | |
| **Sensitivity** | 0.717 | 0.889 | 0.843 | 0.808 | 0.712 | 0.895 |
| **Specificity** | 0.820 | 0.806 | 0.905 | 0.929 | 0.807 | 0.829 |
| **PPV** | 0.776 | 0.696 | 0.878 | 0.913 | 0.755 | 0.739 |
| **NPV** | 0.769 | 0.936 | 0.877 | 0.839 | 0.769 | 0.936 |
| **Accuracy** | 0.772 | 0.833 | 0.877 | 0.870 | 0.763 | 0.852 |
| **Kappa** | 0.540 | 0.650 | 0.751 | 0.739 | 0.520 | 0.690 |
| **F1 score** | 0.745 | 0.781 | 0.860 | 0.857 | 0.733 | 0.810 |
| *Postmenstrual Age 37 weeks* | | | | | | |
| **Sensitivity** | 0.897 | 0.816 | 0.873 | 0.850 | 0.875 | 0.810 |
| **Specificity** | 0.917 | 0.750 | 0.886 | 0.929 | 0.912 | 0.917 |
| **PPV** | 0.959 | 0.886 | 0.945 | 0.971 | 0.959 | 0.971 |
| **NPV** | 0.805 | 0.632 | 0.756 | 0.684 | 0.756 | 0.579 |
| **Accuracy** | 0.904 | 0.796 | 0.877 | 0.698 | 0.886 | 0.833 |
| **Kappa** | 0.785 | 0.537 | 0.725 | 0.698 | 0.743 | 0.601 |
| **F1 score** | 0.927 | 0.850 | 0.908 | 0.906 | 0.915 | 0.883 |
| *Postmenstrual Age 40 weeks* | | | | | | |
| **Sensitivity** | 0.698 | 0.625 | 0.821 | 0.647 | 0.759 | 0.647 |
| **Specificity** | 0.824 | 0.773 | 0.879 | 0.850 | 0.839 | 0.850 |
| **PPV** | 0.830 | 0.800 | 0.868 | 0.880 | 0.830 | 0.880 |
| **NPV** | 0.689 | 0.586 | 0.836 | 0.586 | 0.771 | 0.586 |
| **Accuracy** | 0.754 | 0.685 | 0.851 | 0.722 | 0.798 | 0.722 |
| **Kappa** | 0.512 | 0.379 | 0.701 | 0.455 | 0.597 | 0.455 |
| **F1 score** | 0.758 | 0.702 | 0.844 | 0.746 | 0.793 | 0.746 |
| PPV: positive predictive value; NPV: negative predictive value | | | | | | |
